# The causal relationship between gut microbiota and lipid metabolism in heart failure: A 2-sample Mendelian randomization study

**DOI:** 10.1097/MD.0000000000045087

**Published:** 2025-10-31

**Authors:** Junhong Gan, Naiqiang Hu, Junyao Jiao, Shanliang Li, Jianxiang Li, Zhuo Zhang, Lin Xu, Changxuan Li, Jian Li, Guihua Yue

**Affiliations:** a Department of Critical Care Medicine, Guangxi International Zhuang Medicine Hospital Affiliated to Guangxi University of Chinese Medicine, Nanning, Guangxi, China; b Department of Pulmonary Diseases, Guangxi International Zhuang Medicine Hospital Affiliated to Guangxi University of Chinese Medicine, Nanning, Guangxi, China; c Department of Endocrinology and Metabolism, Guangxi International Zhuang Medicine Hospital Affiliated to Guangxi University of Chinese Medicine, Nanning, Guangxi, China; d Department of Pharmaceutics, School of Pharmacy, Guangxi University of Chinese Medicine, Nanning, Guangxi, China; e Department of Cardiology, Affiliated Ruikang Hospital of Guangxi University of Chinese Medicine, Nanning, Guangxi, China; f Department of Interventional Cardiology, Guangxi International Zhuang Medicine Hospital Affiliated to Guangxi University of Chinese Medicine, Nanning, Guangxi, China; g Department of Neurology, Guangxi International Zhuang Medicine Hospital Affiliated to Guangxi University of Chinese Medicine, Nanning, Guangxi, China; h Graduate School, Guangxi University of Chinese Medicine, Nanning, Guangxi, China; i Department of Cardiology, Affiliated International Zhuang Medicine Hospital of Guangxi University of Chinese Medicine, Nanning, Guangxi, China; j Guangxi University of Chinese Medicine, Nanning, Guangxi, China.

**Keywords:** gut microbiota, heart failure, lipid metabolism, Mendelian randomization

## Abstract

Heart failure is a major global health burden with increasing incidence and mortality. Emerging evidence suggests that gut microbiota (GM) and lipid metabolism may play key roles in heart failure, but their causal relationships remain unclear. We performed a 2-sample Mendelian randomization (MR) analysis using genome-wide association study data from European cohorts to investigate the causal effects of GM on heart failure. To assess mediation, a 2-step MR and multivariable MR were applied to quantify the role of 179 lipid metabolites. Robustness of findings was evaluated through multiple sensitivity analyses. Significant causal associations were observed between several GM taxa (e.g., *Bifidobacterium catenulatum, Lawsonibacter sp000492175*) and heart failure. Multiple lipid metabolites, particularly phosphatidylcholine (PC) subtypes, were identified as mediators, with mediation proportions ranging from 7% to 13%. Sensitivity analyses supported the stability of the results. This study provides evidence for a causal pathway linking GM to heart failure through lipid metabolism. The findings highlight potential microbiota-based and metabolic intervention targets, offering new insights into heart failure pathogenesis and informing precision prevention strategies.

## 1. Introduction

In recent decades, heart failure (HF) has become a major global health challenge, with an estimated 64 million individuals affected worldwide. It is characterized by high morbidity, poor prognosis, and growing healthcare costs, particularly in aging populations where disease prevalence is disproportionately higher.^[1]^ Although genetic susceptibility, metabolic disorders, and cardiovascular risk factors have been implicated, the underlying mechanisms of heart failure remain incompletely understood.^[2]^

Gut microbiota (GM), as a key component of the human microbiome, has attracted increasing attention for its role in cardiovascular health. Dysbiosis of GM has been linked to multiple diseases, including cardiovascular disease, diabetes, and obesity.^[3]^ Specific taxa such as Firmicutes, Bacteroidetes, Lactobacillus, and Bifidobacterium are essential for maintaining host metabolic and immune balance. GM influences cardiovascular physiology through the production of short-chain fatty acids, regulation of the gut–blood barrier, and modulation of systemic inflammation.^[4,5]^ short-chain fatty acids, in particular, exert anti-inflammatory effects via G protein-coupled receptors (GPR41 and GPR43), regulate lipid and glucose metabolism, and reduce endotoxin translocation, thereby contributing to cardiovascular protection.^[6]^

Lipid metabolites, including fatty acids, triglycerides, and cholesterol, are central mediators of cardiovascular disease, and disturbances in lipid metabolism are closely associated with the onset and progression of HF.^[7–10]^ However, the causal role of GM–lipid metabolism interactions in HF remains poorly understood, and previous observational findings may have been confounded by lifestyle and environmental factors.

Mendelian randomization (MR), which uses genetic variants as instrumental variables (IVs), offers a powerful strategy to strengthen causal inference by minimizing bias from confounding and reverse causation.^[11,12]^ To address the current knowledge gap, this study aims to investigate whether GM contributes to the development of HF through lipid metabolism using a 2-sample MR framework.

## 2. Methods

### 2.1. Research design

**Figure 1.**
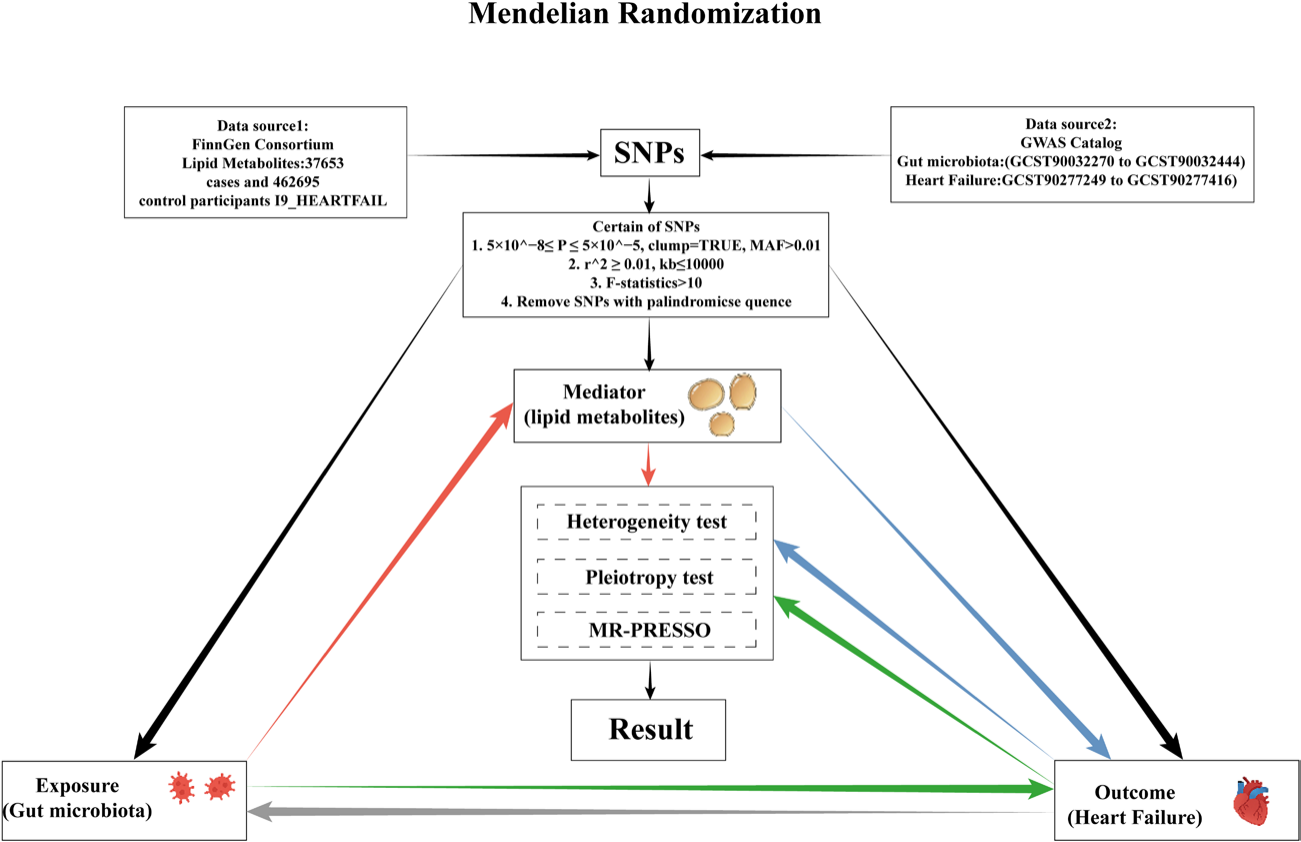
Mendelian randomization framework linking GM, lipid metabolites, and HF. Genetic instruments for GM and heart failure were obtained from the GWAS catalog; lipid metabolite data were from the FinnGen Consortium. SNPs were selected based on clumping and quality filters. The analysis assessed the direct and mediated effects using MR, including heterogeneity, pleiotropy, and MR-PRESSO tests. GM = gut microbiota, GWAS = genome-wide association studies, HF = heart failure, MR = Mendelian randomization, SNPs = single nucleotide polymorphisms.

A 2-sample MR method was employed to investigate the potential causal relationship between GM and HF. To deepen the understanding of the mediation by lipid metabolites, a 2-step MR strategy was adopted. The design and progress of the study were illustrated in Figure 1.

### 2.2. Data sources

GM summary statistics were obtained from a genome-wide association study (GWAS) of 5959 Finnish individuals in the FINRISK 2002 cohort, with matched genotype, fecal metagenome, dietary, and health registry data. In total, 2801 microbial taxa were tested across multiple taxonomic levels, including 11 phyla, 19 classes, 24 orders, 62 families, 146 genera, and 209 species based on the Genome Taxonomy Database (GTDB). Of these, 473 taxa were identified as showing significant associations with host genetic variants. The summary statistics used in this study were downloaded from the GWAS Catalog using the corresponding study accession ID (Table S1, Supplemental Digital Content, https://links.lww.com/MD/Q454).^[13]^

The lipid metabolite dataset contained 179 metabolites spanning 13 categories and 4 major lipid classes: glycerolipids, glycerophospholipids, sphingolipids, and sterols. These metabolites were included according to 3 principles: broad coverage of lipid metabolic pathways, established relevance to cardiovascular and metabolic disorders, particularly HF, and reproducibility and reliability across metabolomics platforms. All study participants were restricted to those of European descent to minimize population stratification bias, and comprehensive demographic and health information was available.^[14]^ Heart failure (HF) summary statistics were extracted from the FinnGen R12 GWAS, comprising 37,653 cases and 462,695 controls, all of European ancestry^[15]^ (Table S1, Supplemental Digital Content, https://links.lww.com/MD/Q454).

This study was conducted exclusively on publicly available GWAS summary statistics. All original studies obtained ethical approval from their respective committees and informed consent from participants. Consequently, no additional ethical approval was required for the present analysis.

### 2.3. SNPs selection

The validity of MR analysis was based on 3 important assumptions: the IV should not be confounded; there should be a strong association between the IV and the exposure; the IV can only affect the outcome through the exposure.

The first step was to select single nucleotide polymorphisms (SNPs) from GWAS summary data that were related to the exposure. Only those exposures that were identified as having a genome-wide significant association with the trait (*P* ≤ 5 × 10^−8^) were selected as IVs. Given the limited number of IVs, the significance level was relaxed to 5 × 10^−5^ to avoid potential errors from the limited SNP pool. Linkage disequilibrium clustering was applied to exclude unwanted specific SNPs (*r*^2^ ≥0.01, window size ≤10,000 kb). Subsequently, the datasets were synchronized for exposure and outcome, and palindromic SNPs with allele frequencies close to 0.5 were eliminated.^[16]^

To ensure the reliability of the exposure genetic tool, the F-statistic was determined using the given formula: *F* = R^2^×[(N − 1 − k)/k] × (1 − R^2^). Here, R² was defined as the total variance explained by the selected SNPs, N was defined as the sample size, and k was defined as the number of SNPs considered. An *F*-statistic higher than ten was considered to indicate sufficient strength, thereby alleviating concerns about weak instrument bias in the 2-sample MR.^[17]^ Because both the GM GWAS (FINRISK 2002) and FinnGen are based on Finnish cohorts, partial sample overlap could not be fully excluded. Such overlap may bias causal estimates toward observational associations. To mitigate this risk, strong genetic instruments (*F*-statistic >10) were used, and sensitivity analyses – including leave-one-out analysis and heterogeneity tests – were conducted to ensure that the causal estimates were not driven by a small subset of potentially overlapping individuals.^[18]^

Although MR reduces confounding by exploiting genetic variants as IVs, residual confounding cannot be entirely excluded. Potential confounders include dietary patterns, environmental exposures, medication use (e.g., antibiotics, lipid-lowering drugs, antihypertensives), and comorbid conditions such as diabetes or obesity. Because this study was conducted using summary-level GWAS data, individual-level adjustments were not possible. Therefore, these factors were acknowledged as limitations of the present study.^[19,20]^

### 2.4. Statistical analysis strategy

Two-way 2-sample MR analysis was performed to systematically assess the causal association between GM and HF. The inverse variance weighting method was applied as the core inference method. To ensure the robustness of the results, the weighted median method and MR-Egger regression were supplemented, in which the MR-Egger intercept term was applied to detect potential directional pleiotropy.^[21,22]^

In addition, the MR-PRESSO method was introduced to identify pleiotropy and outliers, and Cochran Q test was used to evaluate data heterogeneity. When significant heterogeneity was detected, abnormal exposure–outcome pairs were excluded and re-analyzed to improve the robustness and credibility of the results.^[17]^ All statistical analyses were performed in R software (version 4.3.1), and the “TwoSampleMR” package was used to achieve data harmonization and to implement multiple MR methods to ensure the consistency of research conclusions. To display the results more intuitively, Python-based visualization tools were applied to generate graphical representations, which clearly presented the findings and improved the interpretation of the results.

## 3. Results

### 3.1. Two-sample MR analysis between GM and heart failure

**Figure 2.**
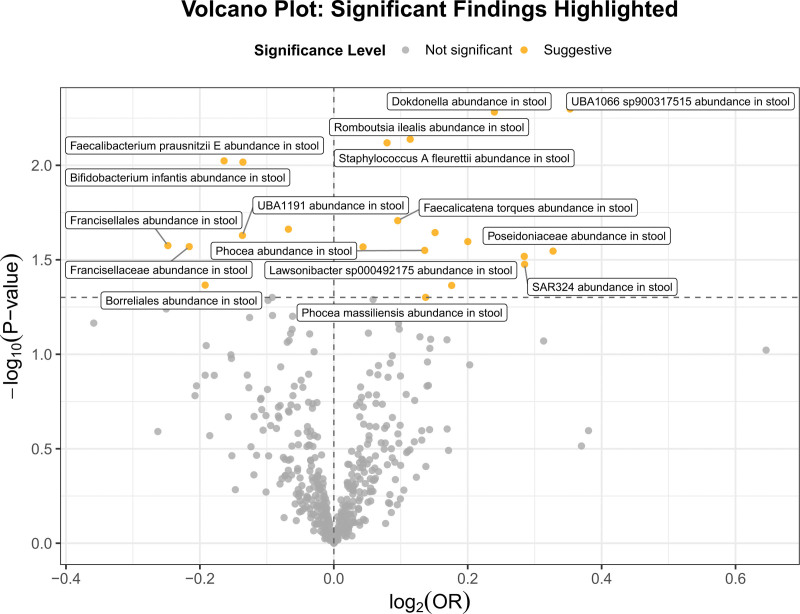
Volcano plot of Mendelian randomization results for GM and heart failure. The plot displays the log₂-transformed odds ratios (x-axis) and–log₁₀ (*P*-values) (y-axis) for the associations between specific gut bacterial taxa and heart failure risk. Each point represents a bacterial trait. Gray dots indicate nonsignificant associations; orange dots indicate suggestive associations. GM = gut microbiota.

The association between specific gut bacterial taxa and heart failure was explored using MR. The volcano plot in Figure 2 illustrates the significance and direction of these associations. Notably, *Faecalicatena torques* was positively associated with heart failure (OR = 1.10; 95% CI: 1.015–1.192; *P* = .020). Conversely, *Bifidobacterium infantis* (OR = 0.873; 95% CI: 0.788–0.968; *P* = .010) and *Bifidobacterium catenulatum* (OR = 0.934; 95% CI: 0.882–0.990; *P* = .022) exhibited protective associations. Other taxa, including *Parabacteroides sp000436495* (OR = 1.045) and *Lawsonibacter sp000492175* (OR = 1.192), were also positively associated with HF, while *Faecalibacterium prausnitzii E, Borreliales*, and *Francisellaceae* demonstrated protective effects. Additional taxa such as *Francisellales, Syntrophorhabdia, Dokdonella*, and *Saccharomonospora* further highlight the complexity of the GM–HF relationship. For comprehensive details, please refer to Table S2, Supplemental Digital Content, https://links.lww.com/MD/Q454.

Sensitivity analyses of 21 gut microbial taxa showed overall robustness. Most taxa had no significant heterogeneity (*Q* test *P* >.05), except for *Faecalicatena torques* and a few others with borderline values (*Poseidoniaceae, Phocea, Phocea massiliensis*). MR-Egger intercepts were mostly nonsignificant, indicating limited pleiotropy. Only *SAR324* showed a marginal effect. MR-PRESSO confirmed these findings, with potential outliers detected in *Poseidoniaceae, Phocea*, and *Phocea massiliensis*. Detailed sensitivity-analysis results of 21 gut microbial taxa are summarized in Table S3, Supplemental Digital Content, https://links.lww.com/MD/Q454, and corresponding visualizations including forest plots, funnel plots, scatter plots, and sensitivity-analysis are provided in PDF S1, Supplemental Digital Content, https://links.lww.com/MD/Q455.

Notably, reverse MR analysis revealed a bidirectional causal relationship between *Faecalicatena torques* abundance in stool and HF (Table S4, Supplemental Digital Content, https://links.lww.com/MD/Q454).

### 3.2. Screening of mediators

**Figure 3.**
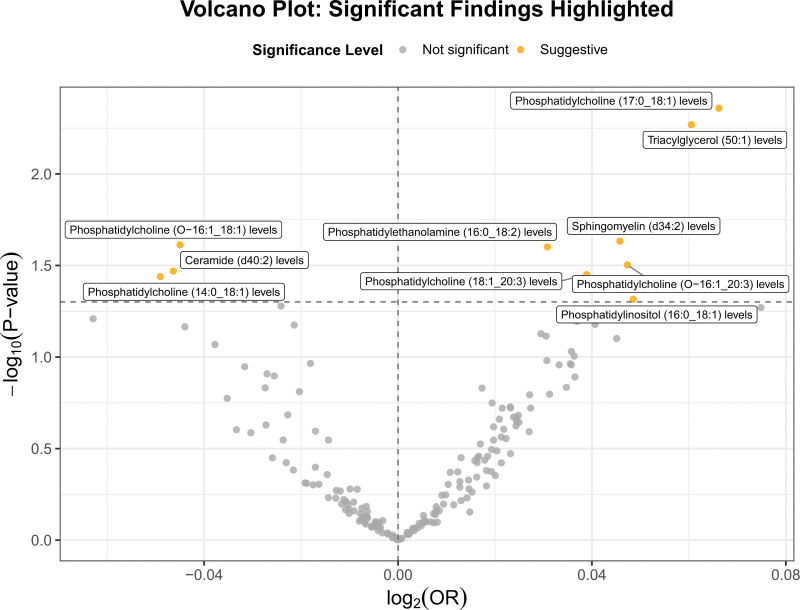
Volcano plot of associations between lipid metabolites and heart failure. This plot visualizes the Mendelian randomization results for 179 lipid metabolites tested as potential mediators of heart failure. The x-axis shows log_2_ (OR), and the y-axis shows–log₁₀ (*P*-value). Orange dots highlight suggestive associations.

To identify potential mediators, 179 lipid metabolites were selected to study their effects on HF. In the analysis of the association between these lipid metabolites and HF, ten important causal relationships were found (Table S5, Supplemental Digital Content, https://links.lww.com/MD/Q454, Fig. 3). Phosphatidylcholine (PC) (14:0_18:1) levels (OR = 0.952; CI = 0.909–0.997; *P* = .036), Ceramide (Cer) (d40:2) levels (OR = 0.955; CI = 0.915–0.996; *P* = .034), PC (O-16:1_18:1) level (OR = 0.956; CI = 0.919 0.994; *P* = .024) were associated with reduced risk. Notably, PC (18:1_20:3) levels(OR = 1.04; CI = 1.003–1.078; *P* = .035), PC(O-16:1_20:3) levels (OR = 1.048; CI = 1.004 1.095; *P* = .031), PC (17:0_18:1) levels (OR = 1.068; CI = 1.021 1.118; *P* = .004) were positively correlated with HF, suggesting that different subtypes of PC may play different roles in the pathophysiological process of HF. Again, phosphatidylethanolamine (PE) (16:0_18:2) levels, Sphingomyelin (d34:2) levels, phosphatidylinositol (16:0_18:1) levels and Triacylglycerol (50:1) levels were associated with odds ratios of 1.031 (*P* = .025), 1.047 (*P* = .023), 1.05 (*P* = .048) and 1.062 (*P* = .005). The above findings provide evidence for lipid metabolites as early biomarkers of HF, providing a basis for further mediation analysis.

To verify the robustness of the identified lipid mediators between GM and HF, sensitivity analyses were performed using Cochran *Q* test, MR-Egger regression, and the MR-PRESSO global test. Most lipid metabolites showed no significant heterogeneity or pleiotropy (*P* >.05), indicating stable and reliable causal estimates. Slight heterogeneity was observed for Ceramide (d40:2) and PC (O-16:1_20:3), and MR-PRESSO detected potential outliers only for Ceramide (d40:2) (*P* = .036). All MR-Egger intercepts were nonsignificant, suggesting no strong evidence of directional pleiotropy. Detailed results are provided in Table S6, Supplemental Digital Content, https://links.lww.com/MD/Q454, and corresponding visualizations can be found in PDF S2, Supplemental Digital Content, https://links.lww.com/MD/Q455.

**Figure 4.**
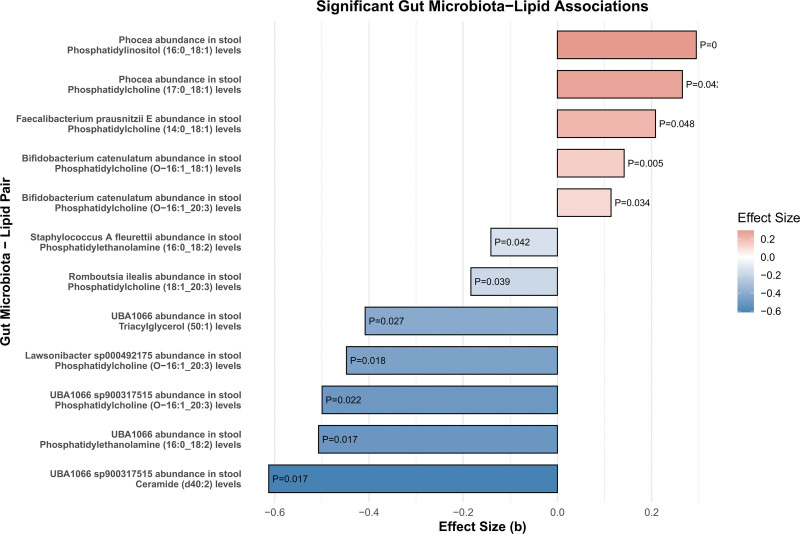
Significant associations between GM and lipid metabolites. This bar plot shows the effect sizes (b) and *P*-values for significant associations between gut microbial taxa and specific lipid metabolites. These associations were identified as potential mediators in the causal pathway from GM to heart failure. The color gradient represents the direction and magnitude of effect sizes. GM = gut microbiota.

In addition to the analysis of the effect of lipid metabolites on HF, this study further explored the potential mediating effect of GM exposure on these important mediators (Table S7, Supplemental Digital Content, https://links.lww.com/MD/Q454, Fig. 4). *Bifidobacterium catenulatum, Lawsonibacter sp000492175* and *UBA1066 sp900317515* affect HF by affecting PC (O-16:1_20:3) levels. The effect sizes were 0.114, −0.448 and −0.500 (*P* = .034, *P* = .018 and *P* = .022, respectively). In addition, *Bifidobacterium catenulatum* was mediated by PC (O-16:1_18:1) levels (β = 0.141, *P* = .005), *UBA1066 sp900317515* was mediated by Cer (d40:2) levels (β = −0.613, *P* = .017). *Phocea* demonstrated a significant mediating effect on HF through 2 different mediators: PC(18:1_20:3) levels (β = 0.265, *P* = .423), Phosphatidylinositol (16:0_18:1) levels (β = 0.295, *P* = .033). In addition, *UBA1066* exhibited significant mediating effects on HF through 2 different mediators: PE (16:0_18:2) levels (β = −0.507, *P* = .017) and Triacylglycerol (50:1) levels (β = −0.408, *P* = .027). *Faecalibacterium prausnitzii E* showed a mediating effect through PC(14:0_18:1) levels, with an effect size of 0.208 (*P* = .048). *Romboutsia ilealis* and *Staphylococcus A fleurettii* showed a negative mediating effect through PC (18:1_20:3) levels and PE(16:0_18:2) levels. The effect sizes were −0.184 and −0.141 (*P* = .039, *P* = .042). Sensitivity analyses including Cochran *Q* test, MR-Egger regression, and MR-PRESSO global test revealed no evidence of heterogeneity, pleiotropy, or outlier effects, indicating robust and consistent causal estimates. Detailed results are provided in Table S8, Supplemental Digital Content, https://links.lww.com/MD/Q454.

### 3.3. Multivariate MR and mediation analyses

**Figure 5.**
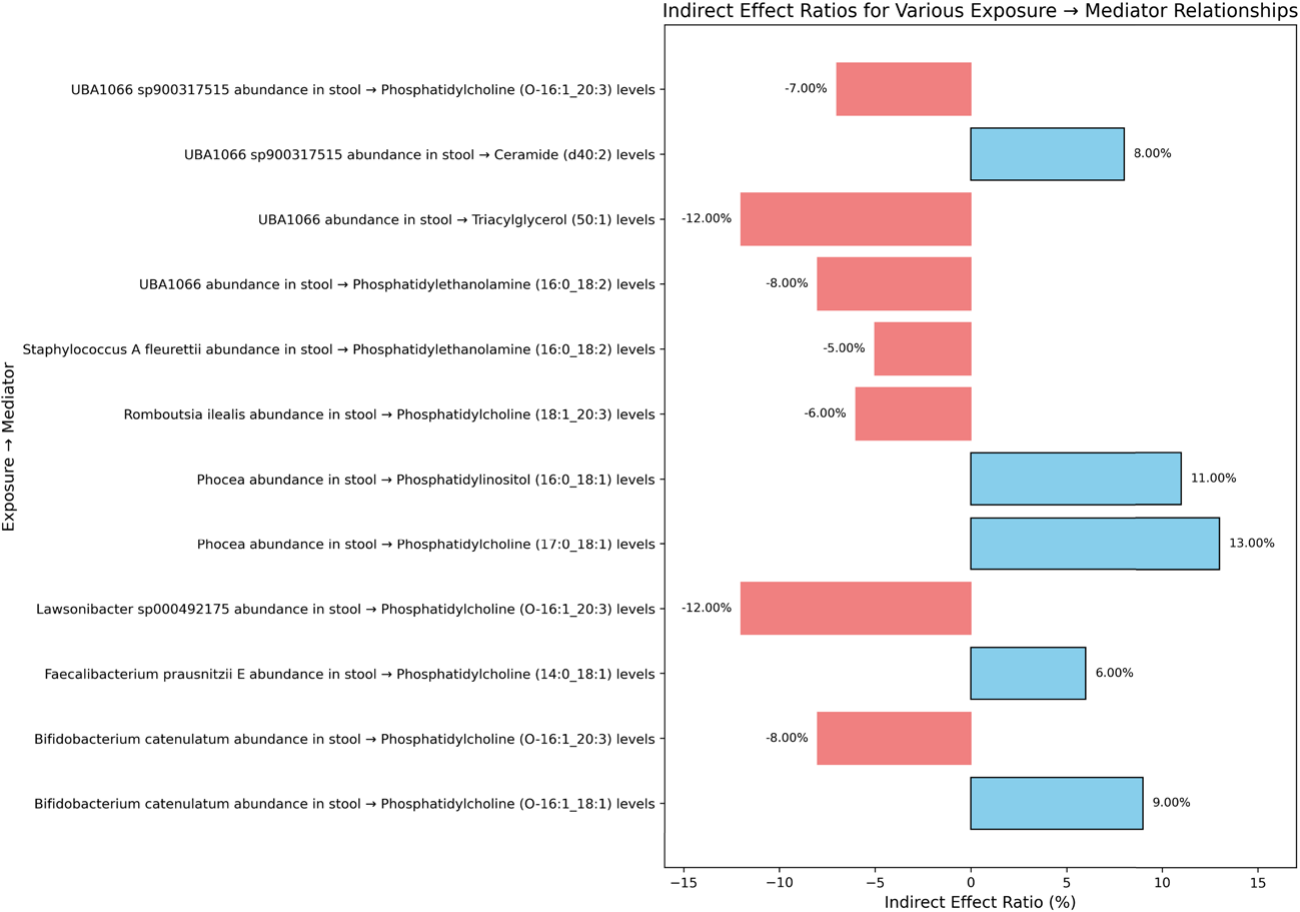
Indirect effect ratios of GM on heart failure mediated by lipid metabolites. This bar plot illustrates the proportion of the effect of specific gut microbial taxa on heart failure that is mediated through individual lipid metabolites. Positive and negative values indicate the direction of the mediation effect. GM = gut microbiota.

After identifying important mediators of HF and subsequent effects of exposure on mediation, the mediation effect proportion was quantified. This required the calculation of indirect effects, derived from the total effect minus the direct effect, and the assessment of direct effects based on the direct effect of GM, adjusted for mediators in the multivariate MR analysis (Table S7, Supplemental Digital Content, https://links.lww.com/MD/Q454, Figs. 4 and 5). To be specific, *Bifidobacterium catenulatum, Lawsonibacter sp000492175 and UBA1066 sp900317515* affected HF by mediating PC (O-16:1_20:3) levels (effect ratio −8.00%, −12.00%, −7.00%). *Bifidobacterium catenulatum* had a 9.00% effect on HF mediated by PC (O-16:1_18:1) levels. The effect of Phocea on HF mediated by Phosphatidylinositol (16:0_18:1) levels was 11%, and the effect of Phocea mediated by PC (17:0_18:1) levels was 13.00%. These ratios highlight the complex dynamics between specific GM exposures, their mediators, and their cumulative effects on HF.

## 4. Discussion

In this study, we provide evidence that specific gut microbial taxa, including *Bifidobacterium catenulatum* and *Lawsonibacter sp000492175*, are causally associated with HF. Furthermore, we identified multiple lipid metabolites – particularly PC subtypes – as key mediators of this relationship, with mediation proportions ranging from 7% to 13%. These findings highlight a novel causal pathway whereby GM influences HF risk through lipid metabolic alterations, offering potential targets for microbiota-based and metabolic interventions.

Our results extend previous observational studies that reported associations between GM dysbiosis, altered lipid metabolism, and cardiovascular disease. Unlike prior work, which was limited by residual confounding and reverse causality, our 2-sample MR design strengthens causal inference. For example, the observed bidirectional effect of *Faecalicatena torques* and the distinct roles of PC subtypes underscore the complexity of GM–lipid–HF interactions, which had not been systematically evaluated before.

A recent MR study by Huang L et al., using MiBioGen data (196 taxa, 16S sequencing), demonstrated causal links between several gut microbial taxa and HF. In contrast, our analysis based on metagenomic data from FINRISK 2002 (473 taxa) highlights lipid metabolites, particularly phosphatidylcholine subtypes, as mediators, thus providing mechanistic insight beyond taxonomic associations.^[23]^

Consistent with previous studies, our results support an important regulatory role for GM on cardiovascular health. The positive association between the genus *Bifidobacterium* and metabolic health has been demonstrated in several studies. *Bifidobacteria* are known to mitigate oxidative stress-induced cardiac injury by enhancing the activity of antioxidant enzymes, such as catalase, and regulating the Nrf2 signaling pathway.^[24,25]^ Studies have shown that metabolites of *Bifidobacterium catenulatum*, such as acetate, can indirectly promote butyrate production, which is known to have anti-inflammatory effects. In addition, *Bifidobacterium* species can reduce the expression of pro-inflammatory factors such as TNF-α and IL-6, and improve the cardiovascular risk caused by inflammation. In animal models, prophylactic administration of the probiotic *Bifidobacterium infantis* was found to protect cardiac function and prevent adverse cardiac remodeling after I/R, and its cardiac protective effect was mainly achieved through the metabolite inosine.^[26]^ These results highlight the importance of GM in regulating systemic metabolism, inflammation, and oxidative stress and further support *Bifidobacterium* genus probiotics as a potential strategy for cardiovascular disease prevention and intervention.

In this study, the abundance of *Romboutsia ilealis* and *Staphylococcus A fleurettii* was positively correlated with HF, while that of *Faecalibacterium prausnitzii* was significantly negatively correlated with HF. *Romboutsia ilealis* is a mixed acid fermentative bacterium with major metabolites including acetic acid, formate, and lactic acid. Acetic acid can be used as a metabolic substrate of myocardium to support energy supply when appropriate. However, in excess, it may lead to acid load and aggravate metabolic disorders. Lactic acid is thought to accumulate in the blood and may trigger lactic acidosis, which is particularly dangerous in patients with advanced HF.^[27]^It has been shown that *S. fleurettii* has a significantly lower internalization capacity compared to other coagulase-negative staphylococci such as *Staphylococcus chromogenes* and *Staphylococcus aureus*. Under specific experimental conditions (when immunity is impaired or infection pressure is increased), *Staphylococcus fleurettii* adhesion and internalization ability increased with time, but it was still lower than other studied strains.^[28]^ In mouse experiments, gavage of *F. prausnitzii* significantly reduced the area of atherosclerotic plaques, indicating significant anti-inflammatory and cardiovascular protective effects. At the same time, *F. prausnitzii* enhanced the mechanical strength of the intestinal barrier and the mucosal protective function by up-regulating the expression of ZO-1 and MUC-2 proteins. In addition, it can inhibit the activity of lipopolysaccharide synthesis pathway, thereby reducing the systemic inflammatory response triggered by endotoxin.^[29]^ This suggests that GM diversity and metabolic balance play a key role in the occurrence and development of HF.

Lipid metabolism is closely related to HF. It has been found that bile acid excess can induce cardiac metabolic reprogramming by inhibiting the expression of key regulators of fatty acid metabolism, such as Pgc1a, leading to cardiac dysfunction. Bile acids promote cardiac metabolism from fatty acid oxidation to glucose metabolism by reducing the metabolic capacity of fatty acid oxidation. This metabolic reprogramming is accompanied by pathological changes such as cardiac hypertrophy, slowing heart rate, and decreased exercise tolerance.^[30]^ It was found that myocardial oxidation of long-chain fatty acids, such as palmitic acid, was significantly reduced in the HF group, while glucose oxidation remained normal. This shift in energy source (metabolic reprogramming) can be seen as a compensatory mechanism to reduce dependence on fatty acid oxidation and alleviate metabolic disorders, but may also exacerbate energy deficits.^[31,32]^ Overall, defects in myocardial lipid metabolism may further affect cardiac function.

Mass spectrometry lipidomic studies have identified molecular lipid signatures of cardiovascular disease, including TAG, PE, PC, and other lipid classes.^[33,34]^ This is consistent with the results of this study, and we supplemented more subtypes. Notably, our analysis identified 5 distinct PC subtypes with heterogeneous effects on HF. Specifically, PC (O-16:1_20:3), PC (18:1_20:3), and PC (17:0_18:1) were positively associated with increased risk of HF, whereas PC (O-16:1_18:1) and PC (14:0_18:1) showed protective associations. These subtype-specific differences underscore the complexity of PC metabolism in cardiovascular health. Previous studies have reported that certain PC molecules containing long-chain polyunsaturated fatty acids, such as PC 42:10, are positively correlated with survival and may serve as prognostic markers in HF, whereas others like PC 32:0 have been linked to increased risk.^[9,35]^ PC helps to package and secrete TAG-rich very low density lipoprotein in the liver. Low PC levels increase TAG synthesis by promoting the nuclear translocation of sterol regulatory element binding protein-1, which activates the transcription of adipogenesis-related genes. However, excessive PC will be metabolized to diglyceride, which is further converted to TAG.^[36]^ High levels of TAG accumulation are considered to be a risk factor for HF, which manifests as myocardial lipid toxicity, leading to mitochondrial dysfunction, oxidative stress and inflammation, and ultimately damaging myocardial function.^[37,38]^ PE is considered to be an early lipid marker of HF.^[39]^ Researches have shown that PE significantly aggravates atrial fibrosis by increasing collagen deposition in atrial tissue and up-regulating the expression of fibrosis markers such as α-SMA.^[40,41]^

Current researches show that lipid metabolism disorder is not only one of the hallmark features of HF, but also directly participates in the pathological processes such as imbalance of myocardial energy metabolism, apoptosis, inflammatory response and myocardial fibrosis. Our mediation analysis identified several lipid metabolites that could be involved in the effect of microbiota on HF. This study found that multiple GM affected HF through different subtypes of PC. *Bifidobacterium catenulatum, Lawsonibacter sp000492175* and *UBA1066 sp900317515* all affect PC (O-16:1_20:3) levels HF, *Romboutsia ilealis* affects HF through PC (18:1_20:3) levels. Studies have shown that PC contains choline structure, which is metabolized by intestinal microorganisms to produce TMA, and TMA is oxidized to TMAO by flavin monooxygenase in the liver.^[42]^ TMAO exacerbates the decline of myocardial function by triggering oxidative stress, inflammation, and endothelial dysfunction, and high levels of TMAO are significantly associated with poor prognosis in patients with HF.^[43–45]^ *UBA1066* and *Staphylococcus A fleurettii* negatively mediate HF by regulating PE (16:0_18:2) levels, and PE levels of specific subtypes are usually elevated in HF patients.^[46,47]^ PE has a key role in a variety of cellular functions, such as the regulation of programmed cell death, autophagy and mitochondrial fusion processes.^[40]^ The reduction of PE levels by microorganisms may impair its role in some pathogenic cellular processes and thus partially alleviate the pathological progression of HF. *Bifidobacterium catenulatum* can negatively mediate HF through 2 different subtypes of PC (O-16:1_20:3) and PC (O-16:1_18:1). *Bifidobacterium* can regulate lipid metabolism through lipoteichoic acid secreted by it, mainly through IGF-1 signaling pathway to affect lipid metabolism, reduce fat accumulation, and improve inflammatory state. This suggests that *Bifidobacterium* may reduce lipid peroxidation and inflammatory factor production by reducing the level of PC isoforms, which has a positive effect on HF.^[48]^

## 5. Strengths of the study

This study is the first to demonstrate a causal pathway whereby GM influences HF through lipid metabolites. Using a 2-sample MR framework with multiple sensitivity analyses, we minimized confounding and reverse causality. The integration of high-quality GWAS and metagenomic data enabled us to identify specific PC subtypes as mediators, with mediation proportions of 7% to 13%. These findings provide mechanistic insights into GM–lipid–HF interactions and highlight potential microbiota- and metabolism-based intervention targets with clinical translational value.

However, this study has limitations. Both the GM GWAS (FINRISK 2002) and FinnGen were based on Finnish populations, so partial sample overlap could not be fully excluded, although we used strong instruments and rigorous sensitivity analyses to mitigate this risk. In addition, Bonferroni correction was not applied to all results, raising the possibility of false positives. Data were mainly from European cohorts, limiting generalizability, and some associations may still be influenced by pleiotropy, unmeasured confounders, or limited SNP strength. Environmental and dietary factors were also not fully accounted for. Heterogeneity across different studies was not assessed, since our analyses were based on single-cohort GWAS summary statistics rather than multi-study meta-analysis.

## 6. Conclusion

Despite its limitations, this study provides a new perspective on the complex relationship between GM, lipid metabolism, and HF by integrating multi-omics data and applying advanced statistical methods. Future studies can further deepen and validate our findings by expanding the coverage of populations, increasing experimental validation and dynamic analysis.

## Acknowledgments

We extend our gratitude to all the investigators and participants who contributed to the genetic association summary data used in this study

## Author contributions

**Conceptualization:** Junhong Gan, Guihua Yue.

**Data curation:** Junhong Gan, Naiqiang Hu, Shanliang Li, Jianxiang Li.

**Formal analysis:** Junhong Gan, Lin Xu.

**Funding acquisition:** Guihua Yue.

**Investigation:** Shanliang Li, Jianxiang Li, Lin Xu.

**Methodology:** Junhong Gan, Zhuo Zhang.

**Project administration:** Jian Li, Guihua Yue.

**Resources:** Lin Xu, Jian Li, Guihua Yue.

**Software:** Junhong Gan, Junyao Jiao, Zhuo Zhang.

**Supervision:** Jian Li, Guihua Yue.

**Validation:** Junyao Jiao, Changxuan Li.

**Visualization:** Junhong Gan, Changxuan Li.

**Writing – original draft:** Junhong Gan.

**Writing – review & editing:** Junhong Gan, Jian Li, Guihua Yue.

## Supplementary Material




