# Supplementary material for: The causal relationship between gut microbiota and lipid metabolism in heart failure: A 2-sample Mendelian randomization study
Source: Medicine (Baltimore). 2025 Oct 31;104(44):e45087. doi: 10.1097/MD.0000000000045087 (PMC12582686; doi:10.1097/MD.0000000000045087)

Supplementary PDF 1.

Visual summary of Mendelian randomization sensitivity analyses for 21 gut microbial taxa. Includes forest plots (effect sizes and 95% CIs), funnel plots (for heterogeneity and asymmetry), scatter plots (MR estimates across multiple methods), and leave-one-out analyses to identify influential SNPs.

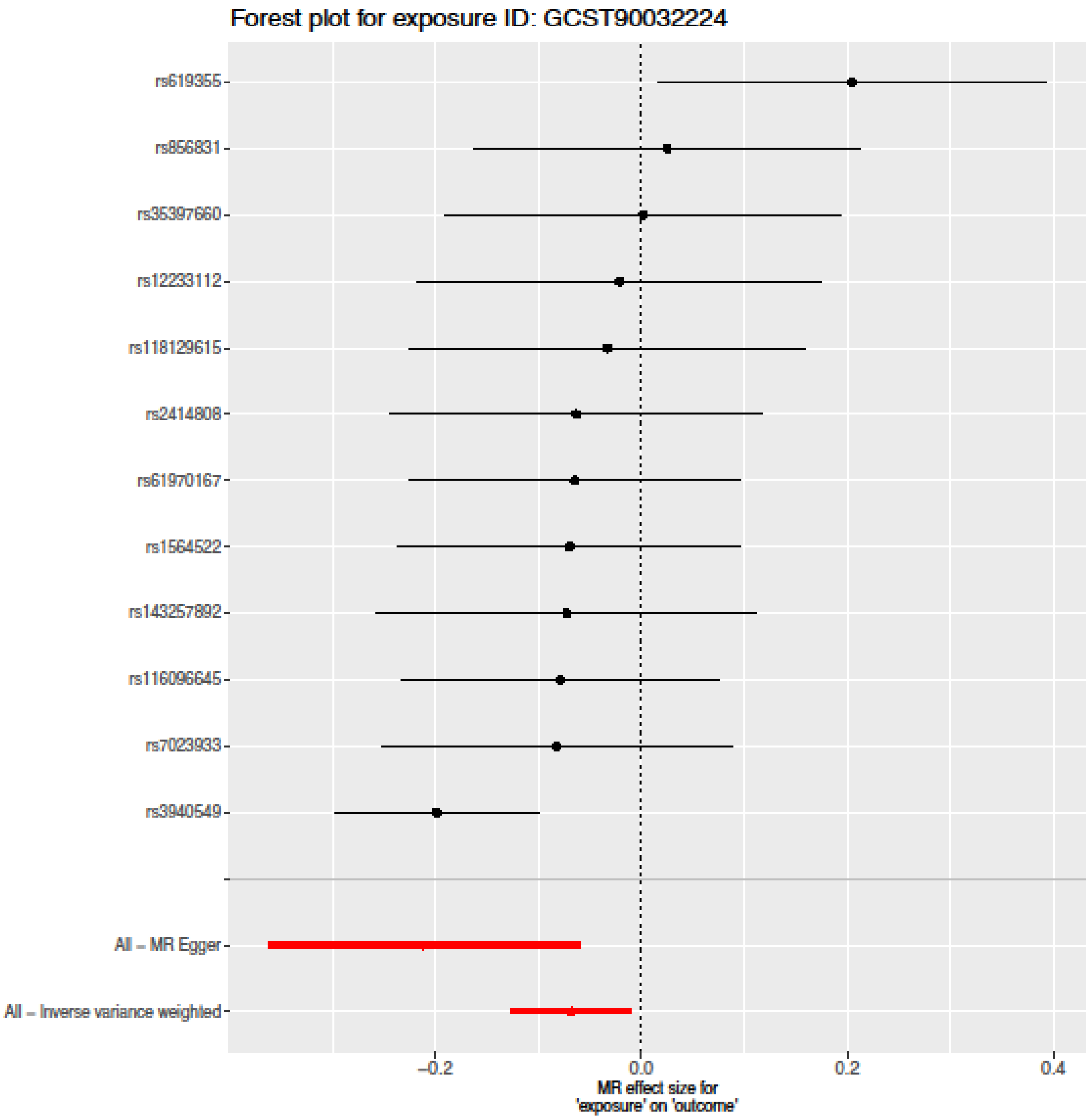

Supplementary PDF 2.

Visualization of MR sensitivity analyses for lipid metabolites. Includes forest plots (effect size per SNP), funnel plots (for heterogeneity assessment), scatter plots (showing consistency across MR methods), and leave-one-out analyses to evaluate the influence of individual SNPs on the causal estimates.

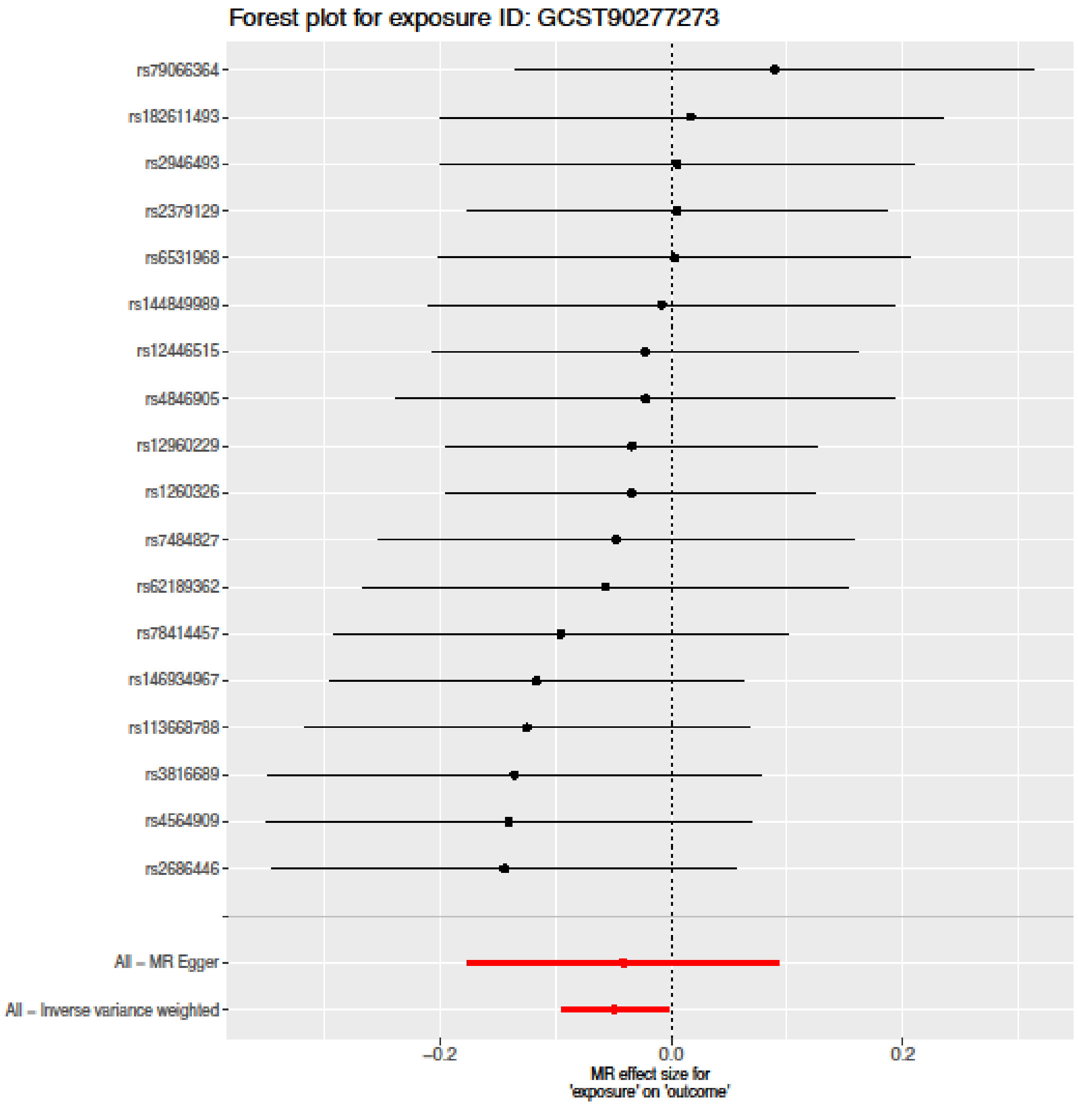

Supplement: Supplementary file 2 [file medi-104-e45087-s002.pdf]
